# A Simple and Improved Method of Taking an Impression with Plaster

**Published:** 1903-05

**Authors:** William H. Potter


A SIMPLE AND IMPROVED METHOD OF TAKING AN IMPRESSION WITH PLASTER. By Dr. E. Latzer and Dr. K. Weisl, Brünn.[1]

The method is as follows: A suitable impression-tray rather large in size is selected. Its upper surface is treated with vaseline and covered with a fine gauze which has been dipped in water. The gauze should not only cover the upper surface of the tray, but extend two or three centimetres over its border. The texture of the gauze should be very delicate, the fabric known in the shops as Meschlin veiling being the most appropriate. The plaster is mixed in the usual way and placed in the impression-tray. When the plaster touches the gauze which covers the upper side of the tray, a certain amount of plaster passes through the meshes of the gauze and lifts it from immediate contact with the tray. It is thus buried in the mass of plaster used in taking the impression. When the proper amount of plaster has been placed in the tray it is carried into the mouth and the plaster allowed to completely harden. The metal tray is now removed, and the fingers are introduced between the cheek and the jaw, and pressure is exerted so as to loosen the impression. Naturally it is broken in many places, but the pieces do not fall into the mouth. They are held together by the delicate mesh of gauze, and the whole mass can be removed from the mouth together. It is then a simple matter to readjust the fragments in their proper position and put the impression back into the tray. The authors of this method have used it in all sorts of cases, including those with pronounced undercuts, and express the greatest satisfaction with the results.

WILLIAM H. POTTER.

[1] Oesterreichisch-Ungarische Vierteljahrsschrift für Zahnheilkunde, Jänner, 1903.

